# Social factors and the prevalence of social isolation in a population-based adult cohort

**DOI:** 10.1007/s00127-021-02174-x

**Published:** 2021-09-17

**Authors:** Susanne Röhr, Felix Wittmann, Christoph Engel, Cornelia Enzenbach, A. Veronica Witte, Arno Villringer, Markus Löffler, Steffi G. Riedel-Heller

**Affiliations:** 1 Institute of Social Medicine, Occupational Health and Public Health (ISAP), Medical Faculty, University of Leipzig, Philipp-Rosenthal-Str. 55, 04103 Leipzig, Germany; 2 Global Brain Health Institute (GBHI), Trinity College Dublin, Dublin, Ireland; 3 Institute for Medical Informatics, Statistics and Epidemiology (IMISE), University of Leipzig, Leipzig, Germany; 4 LIFE—Leipzig Research Center for Civilization Diseases, University of Leipzig, Leipzig, Germany; 5 Max Planck Institute for Human Cognitive and Brain Sciences, Leipzig, Germany; 6 University Hospital Leipzig, Day Clinic for Cognitive Neurology, Leipzig, Germany

**Keywords:** Prevalence, Social isolation, Social exclusion, Epidemiology, Cohort study, Social factors, Socioeconomic status

## Abstract

**Purpose:**

Social isolation has negative effects on physical and brain health across the lifespan. However, the prevalence of social isolation, specifically with regard to sociodemographic and socioeconomic factors, is not well known.

**Methods:**

Database was the Leipzig population-based study of adults (LIFE-Adult Study, *n* = 10,000). The short form of the Lubben Social Network Scale (LSNS-6) was used to assess social isolation (cutoff < 12 points). Sampling weights were applied to account for differences in sampling fractions.

**Results:**

Data were available for 9392 study participants; 51.6% were women, the mean age was 45.2 years (SD = 17.3). The prevalence of social isolation was 12.3% (95% CI 11.6–13.0) across ages 18–79 years. Social isolation was more prevalent in men (13.8%, 95% CI 12.8–14.8) compared to women (10.9%, 95% CI 10.0–11.8; $\chi^{2}$ (1) = 18.83, *p* < .001), and it showed an increase with increasing age from 5.4% (95% CI 4.7–6.0) in the youngest age group (18–39 years) to 21.7% (95% CI 19.5–24.0) in the oldest age group (70–79 years; $\chi^{2}$ (4) = 389.51, *p* < .001). Prevalence differed largely with regard to socioeconomic status (SES); showing lower prevalence in high SES (7.2%, 95% CI 6.0–8.4) and higher prevalence in low SES (18.6%, 95% CI 16.9–20.3; $\chi^{2}$ (2) = 115.78; *p* < .001).

**Conclusion:**

More than one in ten individuals in the adult population reported social isolation, and prevalence varied strongly with regard to sociodemographic and socioeconomic factors. Social isolation was particularly frequent in disadvantaged socioeconomic groups. From a public health perspective, effective prevention of and intervention against social isolation should be a desired target as social isolation leads to poor health. Countermeasures should especially take into account the socioeconomic determinants of social isolation, applying a life-course perspective.

## Introduction

Research consistently shows that social isolation has negative effects on physical health and brain health across the lifespan [1–3]. The hypothesis that social isolation is a chronically stressful condition accelerating aging has found solid supporting evidence since its original formulation over three decades ago [4]. Moreover, social isolation is associated with increased all-cause-mortality [5]. Already early estimates indicated that the detrimental effect of social isolation on health is comparable to that of smoking, obesity or hypertension [6]. A meta-analysis reported that social isolation was associated with increased risk for incident coronary heart disease and stroke [7]. Social isolation has been shown to lead to reports of lower health status, health-related quality of life, and wellbeing, particularly in later life [8, 9]. Studies furthermore indicated that social isolation is detrimental for mental health [10]. It can negatively affect cognitive function and is considered a risk factor for dementia [11, 12].

Despite the robust evidence of social isolation’s health effects, the scientific literature is not without discrepancies, resulting from various definitions, measures and heterogeneous study populations [2]. Social isolation is an objective state defined by minimal contact with others and low levels of community engagement [13]. Social isolation can be understood as the opposite of social integration, i.e., being part of a meaningful social network which provides a resource in times of acute or chronic stress, for example, during illness or loss, and which fosters well-being through regular positive experiences and a stable role in the community [14]. Moreover, social isolation needs to be differentiated from loneliness. Social isolation describes a state of *being* lonely and loneliness refers to a poor subjective evaluation of one owns relationships, hence *feeling* lonely [15]. Individuals can indeed feel lonely despite being integrated in a large social network, and socially isolated individuals may not feel lonely at all [16]. However, social isolation and loneliness tend to be correlated with social isolation rather being a predictor of loneliness than vice versa [17].

The twenty-first century has seen an ongoing debate about a “rise of social isolation” or a “loneliness epidemic” [18], particularly in Western countries. The perception is based on, for example, increasing numbers of single households, reports of low birth rates, and population aging [19, 20]. However, epidemiological data has been conflicting. While some studies concluded that “a general fear” of rising rates of social isolation seems unfounded [21], others described a decline in the average size of social networks over the past 35 years [22]. Moreover, Wrzus et al. (2013) reported that social networks shrink with aging. In light of population aging, which drives a marked increase in the proportion of older individuals, social isolation could indeed increase over time.

The inconclusive picture of social isolation prevalence is also associated with the many indicators used to assess it. Indicators of social isolation vary widely and often comprise proxy measures, such as living alone, being unmarried, and not participating much in social activities [23]. It has been acknowledged that some indicators are rather rough measures, more likely being risk factors for social isolation than social isolation itself [24]. However, it is such indicators that are routinely collected as part of census data, and thus, are used to provide information on social isolation prevalence. Furthermore, studies have been inconclusive about differences in social isolation prevalence with regard to sociodemographic and socioeconomic variables [18].

Therefore, we aimed to investigate the prevalence of social isolation, using a standardized screening measure for social isolation in an adult population, i.e., the Leipzig Population Study of Adults (LIFE-Adult-Study; *n* = 10,000; age range 18–79 years). Moreover, we aimed to inspect differences in social isolation prevalence with regard to sociodemographic and socioeconomic factors. In light of population aging, our results should provide important information for public health.

## Methods

### Study design

The population-based LIFE-Adult Study was designed to investigate major civilization diseases in the adult population of Leipzig, Germany. The sample was drawn from a random age- and gender-stratified list of individuals between age 40 and 79 years from the registry office of Leipzig city. Additionally, 400 more individuals aged 18–39 years were recruited from the same city registry. The recruitment process was continued until the intended age- and sex-stratified sample size, a total of *n* = 10,000 participants, was reached. Pregnancy and insufficient German language skills were the only exclusion criteria. The response rate was 33%. All participants provided written informed consent. Data assessments were carried out from August 2011 to November 2014 at the LIFE study center as part of the University Hospital of Leipzig. The assessments followed a standard operating procedure protocol and were administered by trained study assistants under the supervision of experienced scientists.

The LIFE-Adult Study adheres to the Declaration of Helsinki. The study protocol was approved by the ethics committee of the Medical Faculty of the University of Leipzig. Further study details have been previously described elsewhere [25].

### Sociodemographic and socioeconomic factors

A structured computer-assisted interview on sociodemographic and socioeconomic variables provided information on relevant characteristics of the participants, such as number of persons living in the household (1, 2, 3, 4, ≥ 5), living together with spouse (yes, no), employment status (employed, unemployed, retired), education (low, middle, high; classified according to the CASMIN classification [26], occupational status, and net equivalence income (NEI; grouped into quartiles). Occupational status was operationalized as a household characteristic, i.e., the professional status of study respondents was compared to that of the principal earner of the household, and the higher value was assigned, respectively. Assigned values for occupational status ranged from 1 to 7, representing professional categories from (1) farmer/unskilled worker/semi-skilled worker to (7) freelance academics/civil servants in highest services/supervisors with ≥ 5 employees, based on the International Socio-Economic-Index of Occupational Status (ISEI) which uses the professional classification ISCO-88 [27]. Education, occupational status and NEI were used to operationalize a socioeconomic status (SES) index according to a validated procedure described in [28], which was classified into low, middle and high SES based on sample distribution.

### Social isolation

To assess social isolation, we administered the short form of the Lubben Social Network Scale (LSNS-6) [29]. The LSNS-6 is a quantitative measure of social network size that assesses the number and frequency of contacts with friends and family as well as social support received by them. Overall, it consists of six items. Three questions concern the frequency of meeting relatives, how many relatives the respondent feels close enough to ask for help, and finally, how many relatives the respondent can talk to about private matters. Three more questions address the same aspects, however, with regard to friends. Each of the equally weighted LSNS-6 items is scored from 0 to 5. The total score ranges from 0 to 30. Higher scores indicate larger social networks. A score below 12 is considered an indicator of social isolation, which means that, on average, there are fewer than two individuals available for the aspects of social networks assessed [29]. Lubben et al. chose lack of redundancy in social relationships as the key criterion for determining and validating the cut point based on previous studies that suggested lack of redundancy was associated with lower social support [29, 30]. The LSNS-6 has good psychometric properties [29].

### Statistical analysis

Sample characteristics were inspected with regard to sociodemographic and socioeconomic variables. Group differences between socially integrated individuals (LSNS-6 ≥ 12) and socially isolated individuals (LSNS-6 < 12) were calculated using Chi-Square tests and *t*-tests, as appropriate.

The prevalence of social isolation was calculated in percent with 95% confidence intervals (CIs) in reference to the total study population with complete LSNS-6 assessments. Prevalence of social isolation was furthermore stratified by sociodemographic and socioeconomic factors as well as cross-stratified by age group, gender and SES. Differences in prevalence with regard to stratification variables were inspected using Chi-Square tests. In addition, we used multivariable logistic regression analysis to test the associations between social isolation and the aforementioned factors (age group: 18–39, 40–49, 50–59, 60–69, 70–79; gender: male, female; number of persons living in the household: 1 to ≥ 5; SES: low, middle, high).

A significance level of *α* = 0.05 (two-tailed) was applied. For all analyses, we adopted sampling weights to account for differences in sampling fractions in the LIFE-Adult-study compared to the general population. Specifically, the sampling weights were calculated based on the Leipzig 2012 population data from the Federal Statistical Office of Germany, using direct standardization in regard to the population stratum proportions of age and gender. Analyses were performed using Stata 16.1 SE software package (StataCorp LLC).

## Results

### Sample characteristics

Complete data of the LSNS-6 were available for 9392 study participants (93.9%); 4850 (51.6%) were women and 4542 (48.4%) were men. The mean age of the study sample was 45.2 years (SD = 17.3). With regard to characteristics of the study sample, socially isolated individuals adversely differed from socially integrated individuals with respect to all sociodemographic factors considered, i.e., they were older, less educated, lived with fewer people in one household, lived less often together with their spouse, had less household income, were more often unemployed or retired, and had more often a lower SES. The mean LSNS-6 score of the study population was 17.6 (SD = 5.1), ranging from 0 to 30. Socially isolated individuals had a mean LSNS-6 score of 8.5 (SD = 2.5) and socially integrated individuals averaged at an LSNS-6 score of 18.8 (SD = 4.0). Table 1 shows detailed results.

**Table 1:** Sample characteristics of the total study sample and with regard to social integration and social isolation (*n* = 9392)

	Total sample	Socially integrated individuals (*n* = 8238)	Socially isolated individuals (*n* = 1154)	Group difference (*p* value)
Age, *M* (SD)	45.19 (17.25)	43.84 (17.05)	54.79 (15.56)	< .001
Female gender, *n* (%)	4851 (51.6)	4332 (52.5)	527 (45.7)	< .001
Education, *n* (%)				< .001
Low	717 (7.6)	545 (6.6)	172 (14.9)	
Middle	5464 (58.2)	4776 (58.0)	687 (59.6)	
High	3203 (34.1)	2910 (35.4)	293 (25.4)	
Number of people in household, *n* (*%*)				< .001
1	2553 (27.2)	2165 (26.3)	389 (33.7)	
2	4259 (45.4)	3665 (44.5)	593 (51.4)	
3	1429 (15.2)	1342 (16.3)	87 (7.5)	
4	914 (9.7)	838 (10.2)	76 (6.6)	
≥ 5	228 (2.4)	221 (2.7)	8 (0.7)	
Living together with spouse, *n* (*%*)	6151 (65.5)	5465 (66.4)	686 (59.5)	< .001
Employment status, *n* (*%*)				< .001
Employed	5877 (62.2)	5376 (65.3)	501 (43.5)	
Unemployed	1534 (16.3)	1307 (15.9)	227 (19.7)	
Retired	1974 (21.0)	1559 (18.8)	425 (36.9)	
Net equivalence income (Euros), *M* (SD)	1620.17 (11,114.51)	1648.10 (1121.51)	1423.32 (1043.31)	< .001
Median	1428.57	1466.67	1266.67	
Socioeconomic status, *n* (%)				< .001
Low	2026 (21.9)	1649 (20.3)	377 (32.7)	
Middle	5501 (58.6)	4851 (59.8)	650 (56.4)	
High	1738 (18.5)	1613 (19.9)	126 (10.9)	
Social engagement*, *M* (SD)	17.55 (5.14)	18.82 (4.00)	8.45 (2.48)	< .001

*Assessed with the short form of the Lubben Social Network Scale (LSSN-6, range 0–30, cutoff for social isolation: < 12)

### Prevalence of social isolation

The population-weighted prevalence of social isolation was 12.3% (95% CI 11.6–13.0) across ages 18–79 years. In total, social isolation was more prevalent in men (13.8%, 95% CI 12.8–14.8) compared to women (10.9%, 95% CI 10.0–11.8; $\chi^{2}$ (1) = 18.83, *p* < 0.001). This pattern persisted across all sociodemographic factors considered (see Table 2 for detailed results), with few exceptions where prevalence did not significantly differ between gender (in the oldest age group/70–79 years; low education; living alone in household; three people living in one household and NEI in the 3rd quartile). Prevalence of social isolation significantly differed by age group, showing an increase with increasing age from 5.4% (95% CI 4.7–6.0) in the youngest age group (18–39 years) to 21.7% (95% CI 19.5–24.0) in the oldest age group (70–79 years; $\chi^{2}$ (4) = 389.51, *p* < 0.001), except for men, who showed a prevalence peak in the age group 60–69 (24.4%, 95% CI 20.5–28.2).

**Table 2:** Prevalence of social isolation in an adult population with regard to sociodemographic factors and stratified by gender (*n* = 9392)

	Total sample		Women (*n* = 4851)		Men (*n* = 4542)		Group difference (*p* value)
	Prevalence (%)	95% CI	Prevalence (%)	95% CI	Prevalence (%)	95% CI	
Overall	12.29	11.63–12.96	10.87	10.00–11.75	13.81	12.81–14.81	< .001
Age group	< .001		< .001		< .001		
18–39	5.35	4.65–6.04	3.97	3.12–4.81	6.78	5.67–7.89	< .001
40–49	12.65	11.14–14.15	11.06	9.01–13.11	14.10	11.92–16.28	.049
50–59	17.89	15.66–20.12	15.75	12.80–18.71	20.16	16.80–23.52	.049
60–69	20.72	18.27–23.16	17.64	14.51–20.77	24.35	20.52–28.18	.008
70–79	21.71	19.45–23.98	20.82	17.88–23.77	22.91	19.37–26.45	.370
Education, *n* (%)	< .001		< .001		< .001		
Low	24.04	20.91–27.81	24.10	19.88–28.32	23.97	19.26–28.68	.986
Middle	12.58	11.70–13.46	11.59	10.41–12.75	13.69	12.37–15.02	.019
High	9.14	8.14–10.14	6.22	5.03–7.42	11.95	10.38–13.53	< .001
Number of people in household, *n* (%)	< .001		< .001		< .001		
1	15.22	13.82–16.61	14.54	12.66–16.43	15.98	13.90–18.05	.323
2	13.93	12.89–14.97	11.61	10.28–12.94	16.46	14.85–18.07	< .001
3	6.05	4.82–7.29	5.79	4.03–7.55	6.29	4.55–8.03	.683
4	8.30	6.51–10.09	6.75	4.48–9.02	9.97	7.16–12.77	.081
≥ 5	3.37	1.01–5.73	1.17	0.01–3.08	6.06	1.37–10.76	.028
Living together with spouse, *n* (%)	< .001		< .001		< .01		
Yes	11.16	10.37–11.94	9.59	8.5–10.64	12.67	11.51–13.84	< .001
No	14.42	13.21–15.63	13.00	11.45–14.55	16.25	14.33–18.17	.009
Employment status, *n* (%)	< .001		< .001		< .001		
Employed	8.52	7.81–9.23	7.11	6.19–8.04	9.95	8.86–11.04	< .001
Unemployed	14.77	12.99–16.55	12.81	10.40–15.21	16.62	14.02–19.22	.034
Retired	21.53	19.71–23.34	19.35	17.06–21.65	24.50	21.58–27.43	.007
Net equivalence income (Euros), *M* (SD)	< .001		< .001		< .001		
1. Quartile (0–1,043.25)	17.22	15.67–18.77	15.49	13.48–17.49	19.32	16.91–21.73	.018
2. Quartile (1,043.26–1,428.57)	14.81	12.73–15.63	11.88	10.07–13.69	17.07	14.71–19.42	< .001
3. Quartile (1,428.58–2000.00)	10.41	9.13–11.69	10.15	8.37–11.94	10.67	8.84–12.50	.695
4. Quartile (≥ 2000.01)	7.99	6.90–9.09	5.44	4.08–6.81	10.12	8.47–11.78	< .001
Socioeconomic status	< .001		< .001		< .001		
Low	18.61	16.92–20.31	16.51	14.32–18.69	21.19	18.53–23.85	< .001
Middle	11.82	10.96–12.67	10.65	9.53–11.77	13.14	11.82–14.41	< .001
High	7.22	6.00–8.44	4.38	2.95–5.82	9.57	7.70–11.44	< .001

Social isolation prevalence showed clear trends with regard to education, NEI, employment status and SES, for both women and men, i.e., higher values in each factor translated in lower prevalence and vice versa. For example, prevalence of social isolation significantly differed according to SES, showing lower prevalence in high SES (7.2%, 95% CI 6.0–8.4) and higher prevalence in low SES (18.6%, 95% CI 16.9–20.3; $\chi^{2}$ (2) = 115.78; *p* < 0.001). Unemployed individuals had higher social isolation prevalence compared to employed individuals (14.8%, 95% CI 13.0–15.6 vs. 8.5%, 95% CI 7.8–9.2), but lower prevalence than in retired individuals (21.5%, 95% CI 19.7–23.3; $\chi^{2}$ (2) = 242.66; *p* < 0.001).

Moreover, social isolation prevalence tended to be lower the more individuals were living together in one household, specifically with 3 or more individuals compared to 1 or 2. Prevalence did not significantly differ between one-person households and two-person households (15.2%, 95% CI 13.8–16.1 vs. 13.9%, 95% CI = 12.9–15.0; $\chi^{2}$ (1) = 2.20; *p* = 0.138). Lastly, social isolation differed between individuals who lived together with their spouse compared to those who did not have a spouse or did not live with their spouse (11.2%, 95% CI 10.4–11.9 vs. 14.4%, 95% CI 13.2–15.6; $\chi^{2}$ (1) = 20.93; *p* < 0.001). The above described differences in social isolation prevalence were also seen in the gender-stratified analysis.

### Prevalence of social isolation stratified by age groups, gender and SES

Further stratifying prevalence of social isolation by age, gender and SES showed that the gender difference in social isolation prevalence persisted across age groups and levels of SES, with men showing higher prevalence than women in all age group and SES categories (Table 3). Moreover, the trend of higher prevalence of social isolation with lower levels of SES was seen across all age groups; however, it was seemingly less pronounced in the youngest age group where men with ages 18–39 years were an exception showing no significant difference in the prevalence of social isolation ($\chi^{2}$ (2) = 0.972; *p* = 0.615). Overall, the prevalence of social isolation tended to increase with age in all levels of SES in men and women alike; again, with one exception: social isolation peaked in men aged 60–69 years and tended to be comparably lower in the oldest age group (70–79 years), specifically with regard to high SES.

**Table 3:** Prevalence (%) of social isolation with corresponding 95% confidence intervals (95% CI) in an adult population stratified by age, gender and socioeconomic status (*n* = 9329)

Age group (years)	Socioeconomic status							
	Men (*n* = 4542)				Women (*n* = 4851)			
	Low% (95% CI)	Middle% (95% CI)	High% (95% CI)	Group difference^1^ (*p* value)	Low% (95% CI)	Middle% (95% CI)	High% (95% CI)	Group difference^1^ (*p* value)
18–39	7.54 (5.08–10.01)	7.17 (5.64–8.71)	6.02 (3.59–8.44)	.615	5.92 (3.96–7.89)	3.69 (2.60–4.78)	2.03 (0.43–3.94)	.013
40–49	33.44 (25.51–41.37)	12.42 (9.81–15.03)	6.82 (3.53–10.12)	< .001	22.40 (15.26–29.53)	11.36 (8.74–13.99)	2.67 (0.42–4.91)	< .001
50–59	37.32 (27.80–46.85)	18.30 (13.99–22.61)	11.07 (5.73–16.41)	< .001	30.37 (21.52–39.21)	15.10 (11.28–18.91)	6.01 (1.99–10.04)	< .001
60–69	39.00 (29.46–48.54)	19.96 (15.19–24.73)	21.54 (13.67–29.40)	< .001	26.37 (18.63–34.11)	16.17 (12.39–19.96)	10.51 (3.47–17.55)	.006
70–79	28.26 (20.13–36.39)	23.92 (19.18–28.66)	13.73 (7.11–20.36)	.034	29.26 (22.66–35.86)	19.17 (15.62–22.73)	9.44 (2.48–16.39)	< .001
Group difference^2^ (*p* value)	< .001	< .001	< .001		< .001	< .001	< .001	

^1^For levels of socioeconomic status in each age group separately for men and women

^2^For age groups in each level of socioeconomic status separately for men and women

**Fig. 1:**
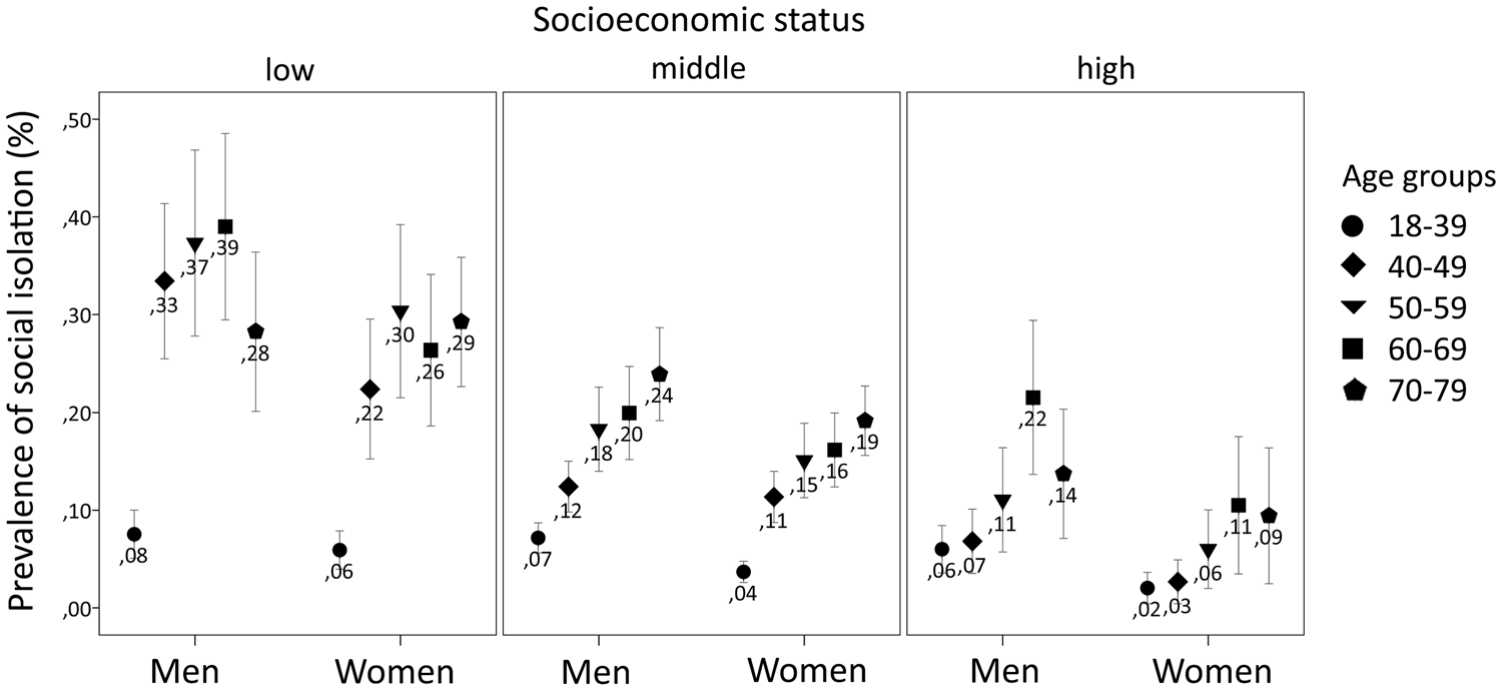
Prevalence of social isolation stratified by age group, gender and socioeconomic status in the LIFE Adult Study (*n* = 9329)

Overall, there was a huge range in the prevalence of social isolation if stratified for age, gender and SES: from 2.0% (95% CI 0.4–3.9%) in women aged 18–39 with high SES to reaching 39.0% (95% CI 29.5–48.5%) in men aged 60–69 with low SES. Figure 1 visually displays social isolation prevalence with respect to age, gender and SES.

### Associations of sociodemographic factors and SES with social isolation

Logistic regression analysis confirmed the associations of age, gender, number of people living together in the household and SES with social isolation in our sample (Table 4). Men were 1.5 times more likely to be socially isolated than women, after adjustment for age and SES (OR 1.5; 95% CI 1.2–1.8; *p* < 0.001). The odds for social isolation increased with age, being particularly pronounced upwards from age 50 years with a more than fourfold likelihood of social isolation in higher age groups compared to ages 18–39 years. Likewise, individuals with low SES were more than three times more likely to be socially isolated compared to individuals with high SES (OR 3.2, 95% CI 2.2–4.4, *p* < 0.001) and almost twice as likely compared to individuals with middle SES (OR 1.8, 95% CI 1.3–2.4, *p* < 0.001).

**Table 4:** Associations between social isolation and sociodemographic factors in an adult population (*n* = 9329)

Social isolation^1^	Odds ratio	95% CI	SE	*p* value
Age group^2^				
40–49	3.15	1.99–5.00	0.74	< .001
50–59	4.25	2.76–6.55	0.94	< .001
60–69	4.66	3.03–7.17	1.02	< .001
70–79	4.70	3.07–7.20	1.02	< .001
Gender^3^				
Male	1.49	1.23–1.80	0.15	< .001
Number of people living in household^4^				
1	2.33	1.56–3.48	0.48	< .001
2	1.78	1.20–2.65	0.36	.004
4	1.49	0.81–3.13	0.55	.179
≥ 5	0.55	0.26–1.15	0.21	.114
Socioeconomic status^5^				
Middle	1.77	1.30–2.40	0.28	< .001
Low	3.15	2.24–4.43	0.55	< .001

^1^Cutoff of < 12 of the total score of the short form of the Lubben Social Network Scale (LSNS-6)

^2^Reference category: age 18–19 years

^3^Reference category: women

^4^Reference category: 3

^5^Reference category: high

## Discussion

We aimed to provide information on the prevalence of social isolation in an adult population with regard to sociodemographic and socioeconomic factors. The overall prevalence of social isolation was 12.3% (95% CI 11.6–13.0%) across ages 18–79 years. Social isolation prevalence was higher in men compared to women, increased with age and strongly varied with regard to socioeconomic factors, with lower status consistently yielding higher prevalence.

Drawing on the scientific literature, there are very few prevalence studies that would allow for result comparison. In a representative sample of 3000 Australian adults, with a similar mean age (45.3 years) to our study, 9% reported some social isolation and 7% reported being isolated or very isolated [31]. Moreover, the authors found comparable prevalence variations by gender, labor force, and income status. Eckhard [24] reported a prevalence of social isolation between 2.0 and 7.8% in individuals aged 18–55 years and between 6.7 and 8.0% for individuals aged 56 years and older in Germany based on three indicators of social isolation (low frequency of social contact with friends, relatives, and neighbors; absence of a discussion network; absence of social support) as assessed using 2011 data of the German Socio-Economic Panel (GSOEP). Based on different data sources from nationally representative samples in the USA, Holt-Lunstad [18] concluded that a significant proportion of the US population, and older adults in particular, may be socially isolated. In the nationally representative Swiss Health Survey 2012, which included individuals aged 15 years and older, 17% of youngsters and adolescents, 20% of young and middle-aged adults, 23% in early late life and 35% or older individuals were only partly integrated or socially isolated according to a multidimensional index of social integration [23]. A paucity of studies on the prevalence of social isolation in the general adult population seems to exist specifically with regard to the utilization of standardized measures to assess social isolation. An exception is the study of social isolation in old age populations. Here, a degree of comparability is reached by the widespread use of the LSNS, which we applied in our study as well. Accordingly, the prevalence of social isolation in urban community-dwelling older adults was reported at 20% in Hamburg (Germany), 11% in the Solothurn (Switzerland) and 15% in London (UK) [29]. Although the LSNS was developed for the use among older individuals, its utility among younger populations has been demonstrated, suggesting it is a valid tool to assess social isolation in the general population [32]. This potential should be leveraged in future studies, as to date, studies that investigate the prevalence of social isolation still largely rely on proxy measures [23].

Our findings showed that men reported being socially isolated more frequently than women, which is in line with previous studies [33, 34]. It has been argued that men tend to have smaller social networks as their social and emotional needs are often met by their spouses or partners, whereas women rely more heavily on multiple sources [35].

Moreover, we found that social isolation increased with aging, being particularly high among older individuals. This is a common finding [21, 36, 37]. With increasing age, decreases in social network size are partially inevitable for varying reasons, such as migration of children, other relatives, and friends, as well as deaths or increasing disabilities of social network members [37]. Interestingly, the rather linear increase of social isolation with aging is different to the pattern of loneliness with regard to aging: Loneliness shows a U-shaped prevalence with increasing age and tends to be less frequent than social isolation, suggesting that older individuals feel less lonely despite often being socially isolated [21].

Notably, the prevalence of social isolation between individuals who lived alone in a household (15.2%) and individuals who lived with one other person (13.9%) did not differ substantially (1.3%). The prevalence only showed a marked difference from three or more persons living in the household. This questions the validity of using the proportion of single households as a marker for social isolation.

The prevalence of social isolation varied strongly with the socioeconomic factors of interest. The more disadvantaged a person was with regard to education, income, employment or overall SES, the higher was the prevalence of social isolation. Additionally, the difference in social isolation and levels of SES were more pronounced with increasing age, while revealing a consistent gender gap to the disadvantage of men. For example, among women aged 18–39 years with a high SES, prevalence of social isolation was 2% and among men aged 60–69 years with a low SES, it was 39%. It is well known that social isolation is a results of a complex interplay between socioeconomic power and inequalities [38]. There is a strong association of those being marginalized having a greater likelihood of experiencing social isolation—our results support this notion. Wealth plays a dominant role in shaping living conditions and physical environments which provide access and opportunities to develop and maintain social connections [39]. Inequities in social connections arise as early as from education in the early lifespan with lower levels of education being consistently associated with higher prevalence of social isolation. Therefore, more equitable chances for good education may improve social inclusion, and hence, mitigate subsequent health disparities of social isolation.

From a public health perspective, it should be of interest to find and implement measures that reduce social isolation in the population, especially in old age, to prevent or attenuate the associated adverse health outcomes. However, current approaches to intervene against social isolation were not overly effective [40, 41]. The same has been reported for evidence regarding the prevention of social isolation in old age [42]. Therefore, it has been suggested to shift to a life-course perspective to identify and prevent social isolation at key life stages—the earlier the better [42]. In light of our study results, we argue that such a focus on prevention must necessarily address the socioeconomic determinants of social isolation to be successful.

### Strengths and limitations

A strength of the study was the large sample covering a good age range that allowed for distinct stratified prevalence estimation. Moreover, applying census-based sampling weights helped nearing representative prevalence estimates of social isolation in a German urban area. Estimates may differ in rural or more remote areas, which could be subject to future studies. Another strength was that our results were based on an established standardized screening instrument for social isolation instead of being derived from widely used proxy measures. This also allowed for comparisons with other studies utilizing the LSNS.

Regarding limitations, however, it needs to be noted that the LSNS-6 is not a diagnostic instrument. It is validated to screen for social isolation based on a quantifying approach of social networks that uses a cutoff score to differentiate between social isolation and social integration. Hence, it is a risk assessment which does not measure qualitative aspects of social ties, such as perceived isolation. This would require clinical and/or more comprehensive assessments, which could thus yield different prevalence estimates. Moreover, generalizability may be compromised by the participation rate of 33% in the LIFE Adult Study [25], which testified to the general trend of declining willingness to participate in epidemiological studies [43]. Moreover, it has to be considered, that the area of study conduction, Leipzig city, was part of the former German Democratic Republic. Special circumstances prevailed after the country breakdown in 1989 may entail a lack of social ties due to escape and social crisis [44]. From that perspective, social isolation may be overestimated in our study. Study participants in the LIFE Adult study included up to 79 years of age. Therefore, our study lacks information on social isolation prevalence in the oldest-old, which has been suggested to be particularly high [11].

### Conclusion

More than one in ten individuals in the adult population reported social isolation, and its prevalence strongly varies with regard to sociodemographic and socioeconomic factors. Social isolation is particularly frequent in disadvantaged socioeconomic groups, and this pattern intensifies with increasing age. From a public health perspective, effective prevention of and intervention against social isolation should be a desired target as social isolation leads to poor physical and brain health. Countermeasures should especially take into account the socioeconomic determinants of social isolation, applying a life-course perspective.

## Data Availability

Data used in this study can be made available to researchers on request to the correspondent author.
